# The Philadelphia Lung Cancer Learning Community: a multi–health-system, citywide approach to lung cancer screening

**DOI:** 10.1093/jncics/pkad071

**Published:** 2023-09-15

**Authors:** Julie A Barta, Cherie P Erkmen, Christine S Shusted, Ronald E Myers, Chelsea Saia, Sarah Cohen, Jocelyn Wainwright, Charnita Zeigler-Johnson, Farouk Dako, Richard Wender, Gregory C Kane, Anil Vachani, Katharine A Rendle

**Affiliations:** Department of Medicine, The Jane and Leonard Korman Respiratory Institute, Division of Pulmonary and Critical Care Medicine, Thomas Jefferson University, Philadelphia, PA, USA; Department of Thoracic Medicine and Surgery, Lewis Katz School of Medicine, Temple University, Philadelphia, PA, USA; Department of Medicine, The Jane and Leonard Korman Respiratory Institute, Division of Pulmonary and Critical Care Medicine, Thomas Jefferson University, Philadelphia, PA, USA; Department of Medical Oncology, Division of Population Science, Thomas Jefferson University, Philadelphia, PA, USA; Department of Family & Community Medicine, University of Pennsylvania, Philadelphia, PA, USA; Department of Family & Community Medicine, University of Pennsylvania, Philadelphia, PA, USA; Department of Family & Community Medicine, University of Pennsylvania, Philadelphia, PA, USA; Department of Medical Oncology, Division of Population Science, Thomas Jefferson University, Philadelphia, PA, USA; Cancer Prevention and Control Program, Fox Chase Cancer Center, Philadelphia, PA, USA; Department of Radiology, University of Pennsylvania, Philadelphia, PA, USA; Department of Family & Community Medicine, University of Pennsylvania, Philadelphia, PA, USA; Department of Medicine, The Jane and Leonard Korman Respiratory Institute, Division of Pulmonary and Critical Care Medicine, Thomas Jefferson University, Philadelphia, PA, USA; Department of Medicine, Division of Pulmonary, Allergy and Critical Care, University of Pennsylvania, Philadelphia, PA, USA; Department of Family & Community Medicine, University of Pennsylvania, Philadelphia, PA, USA

## Abstract

**Background:**

Lung cancer screening uptake for individuals at high risk is generally low across the United States, and reporting of lung cancer screening practices and outcomes is often limited to single hospitals or institutions. We describe a citywide, multicenter analysis of individuals receiving lung cancer screening integrated with geospatial analyses of neighborhood-level lung cancer risk factors.

**Methods:**

The Philadelphia Lung Cancer Learning Community consists of lung cancer screening clinicians and researchers at the 3 largest health systems in the city. This multidisciplinary, multi-institutional team identified a Philadelphia Lung Cancer Learning Community study cohort that included 11 222 Philadelphia residents who underwent low-dose computed tomography for lung cancer screening from 2014 to 2021 at a Philadelphia Lung Cancer Learning Community health-care system. Individual-level demographic and clinical data were obtained, and lung cancer screening participants were geocoded to their Philadelphia census tract of residence. Neighborhood characteristics were integrated with lung cancer screening counts to generate bivariate choropleth maps.

**Results:**

The combined sample included 37.8% Black adults, 52.4% women, and 56.3% adults who currently smoke. Of 376 residential census tracts in Philadelphia, 358 (95.2%) included 5 or more individuals undergoing lung cancer screening, and the highest counts were geographically clustered around each health system’s screening sites. A relatively low percentage of screened adults resided in census tracts with high tobacco retailer density or high smoking prevalence.

**Conclusions:**

The sociodemographic characteristics of lung cancer screening participants in Philadelphia varied by health system and neighborhood. These results suggest that a multicenter approach to lung cancer screening can identify vulnerable areas for future tailored approaches to improving lung cancer screening uptake. Future directions should use these findings to develop and test collaborative strategies to increase lung cancer screening at the community and regional levels.

Annual lung cancer screening using low-dose computed tomography (CT) for individuals at high risk is a critical public health intervention for reducing lung cancer mortality (1-3). Despite major efforts over the past decade to expand lung cancer screening availability and eligibility, lung cancer screening uptake ranges from just 4% to 15% across the United States and in many states is discordant from state-level lung cancer mortality rates (4). National analyses of lung cancer screening facilities have demonstrated that screening accessibility varies by geographic distance, between states and regions, and across rural-urban environments (5-8). One study noted that the vast majority (81.4%) of lung cancer screening–eligible individuals between the ages of 50 and 80 years reside within metropolitan counties, and nearly 10 million eligible individuals in the United States live within an urban core (6). These studies and others demonstrate that geospatial analyses can be used to characterize geographic areas on the basis of disease incidence and accessibility of health-care services as well as socioeconomic factors and social determinants of health (5). Given the current low rates of lung cancer screening uptake, identifying geographic, socioeconomic, racial, and other disparities is a crucial first step for future development of strategies to overcome barriers to lung cancer screening (9). As part of this effort, our analysis juxtaposes the citywide geographic distribution of screening participants with neighborhood-level lung cancer risk factors.

National efforts to evaluate lung cancer screening eligibility and uptake are important for their potential impacts on US health policy and guideline development. They are limited, however, in their capacity to identify community-level barriers and are largely disconnected from local approaches to improving disparities. The Philadelphia Lung Cancer Learning Community (PLC2) has the overarching goal of bringing together local clinical leaders and researchers to develop and implement effective ways to increase lung cancer screening and tobacco treatment specifically in populations at greatest risk (10). Philadelphia has the highest smoking rate among large US cities, with 20% of adults reporting current smoking status in 2019 (11,12). Philadelphia is also one of the poorest large cities, with 23% of residents in a household with an income below the federal poverty level and 11% of adults with no insurance coverage (12). It is estimated that more than 89 000 individuals in Philadelphia may be eligible for lung cancer screening based on the United States Preventive Services Task Force (USPSTF) 2021 recommendation, with the greatest absolute number of eligible people residing in the Upper North and South planning districts and the highest eligibility percentages in the River Wards and North Delaware planning districts (2,13). These 4 planning districts also have high levels of socioeconomic deprivation and increased lung cancer risk across several measures. For example, the River Wards have the highest smoking prevalence (38.8%) among all Philadelphia planning districts, and 25.7% of adults residing there report forgoing health care because of cost within the past year (14). Among these 4 planning districts, the percentage of Philadelphians living in poverty ranges between 19.1% and 29.3%, and up to 15.8% of adults have no health insurance (14).

Although individual institutions in Philadelphia have described lung cancer screening implementation strategies and outcomes in single-center analyses, no studies integrate cohorts across local health systems (15-25). The objectives of this study were to characterize lung cancer screening uptake among the 3 largest health systems in the city of Philadelphia and to integrate census tract-level analyses of lung cancer risk burden. Identification of local neighborhoods experiencing a high burden of lung cancer risk factors, combined with underscreening, can direct future development of targeted, community-based efforts to improve lung cancer screening uptake.

## Methods

### The PLC2

PLC2 health systems include Jefferson Health, Penn Medicine, and Temple Health. The multidisciplinary team consists of experts in pulmonary medicine, thoracic surgery, radiology, primary care, population science, and implementation science and health-care delivery. The PLC2 has an overarching mission of decreasing lung cancer morbidity and mortality across the greater Philadelphia region. The group has met monthly since 2020 to share strategies for high-quality lung cancer screening, review harmonized data, and plan tailored approaches for local lung cancer screening implementation.

The Jefferson Lung Cancer Screening Program is a centralized program with 5 main campuses across the Jefferson Health Enterprise, with screening locations in southeastern Pennsylvania and southern New Jersey. Lung cancer screening programs at each campus are staffed by a nurse practitioner, nurse navigator, and coordinator, who receive electronic referrals and manage all subsequent steps in the lung cancer screening process, including confirming eligibility, performing shared decision making and tobacco treatment counseling, obtaining insurance authorization, scheduling the low-dose CT scan, reviewing lung cancer screening results with patients and referring health-care professionals, evaluating suspicious lung nodules and incidental findings, and tracking patients to maximize annual adherence with screening (15,16). Patient-level data are collected prospectively and recorded in a standardized intake form as part of the Jefferson LCS Registry.

The Penn Medicine Lung Cancer Screening Program is a hybrid program with 5 main campuses that have screening locations in southeastern Pennsylvania and southern New Jersey. Lung cancer screening scans can be ordered by any health system clinician or referred to a centralized lung cancer screening program based at one of the health system hospitals located in Philadelphia that is staffed by a nurse practitioner and coordinator, who receive electronic referrals and manage all subsequent steps in the lung cancer screening process, including confirmation of eligibility, shared decision making and referral to tobacco treatment counseling, insurance authorization, low-dose CT scan scheduling, review of lung cancer screening results with patients and referring health-care professionals, evaluation of suspicious lung nodules and incidental findings, and tracking patients to maximize annual adherence with screening (24).

The Temple Health Lung Cancer Screening Program is a centralized program in north Philadelphia with 3 screening locations. Clinicians can refer patients or submit an order for lung cancer screening. Program nurses receive referrals and orders, contact patients to confirm eligibility, coordinate insurance and payment plans, enter demographic data into a custom data form in the electronic health record (EHR), and arrange an integrated lung cancer screening visit. A physician or advanced practitioner conducts the integrated lung cancer screening visit, which consists of shared decision making, a low-dose CT scan, reporting of results to the patient, coordination of follow-up care, smoking cessation counseling, and data entry into the custom EHR. Outcomes, including Lung CT Screening Reporting & Data System (Lung-RADS) category, diagnostic workup, cancer diagnosis, and treatment of all patients, are reviewed and updated monthly (19-21).

### Study population

The study population for this analysis consisted of individuals undergoing lung cancer screening at Jefferson Health, Penn Medicine, or Temple Health between January 1, 2014, and December 31, 2021. Only lung cancer screening–eligible individuals with a home address within Philadelphia city limits at baseline low-dose CT scan were included in the study. Lung cancer screening eligibility was determined by USPSTF 2021 criteria, prioritizing age and smoking status. Ineligible individuals were defined as those reporting never-smoking status or whose smoking status was never assessed, those with passive cigarette smoke exposure only, and those younger than 50 years of age or older than 80 years of age. Baseline sociodemographic data, Philadelphia census tract of residence, and screening results for baseline and subsequent low-dose CT scans were extracted and harmonized across the 3 health systems. Institutional review board approval was obtained at each PLC2 member health system (Jefferson Control No. 17D.150; Penn Protocol No. 830184; Temple Protocol No. 23095). Individuals who were ineligible for lung cancer screening on the basis of USPSTF 2021 criteria, prioritizing age and smoking status, were excluded from the analysis (26). A collaborative research and data sharing agreement across the 3 health systems was signed on July 19, 2021.

Individuals screened at Jefferson’s Center City or Northeast campuses were identified through the Jefferson LCS Registry, maintained prospectively in Research Electronic Data Capture (2 screening sites in Center City and South Philadelphia) or through the Nuance PowerScribe database (3 sites in northeast Philadelphia). Baseline and subsequent low-dose CT classifications were assigned at the time of data entry in the registry.

Patients screened at Penn Medicine were identified by having a completed lung cancer screening low-dose CT scan at any Penn Medicine facility documented in the EHR using lung cancer screening–specific *Current Procedural Terminology* or *Healthcare Common Procedure Coding System* codes (71271 or G0297, respectively). Body mass index, smoking status, and pack-years were determined using the most recent documented record before or on the baseline lung cancer screening date. Codes used for chronic obstructive pulmonary disease diagnosis on or before baseline lung cancer screening included J44* *(International Statistical Classification of Diseases, Tenth Revision);* 490*, 491*, 492*, 493*, 494*, 495*, 496* *(International Classification of Diseases, Ninth Revision).* For determination of personal history of lung cancer, cancer registry data were complete through 2019. The first completed lung cancer screening was categorized as the baseline low-dose CT scan for each screened patient; screens completed outside of Penn Medicine were excluded.

Patients with documentation of *Current Procedural Terminology* code 71271 or *Healthcare Common Procedure Coding System* code G0297 and screening at Temple’s main campus or 1 of 2 satellite campuses within city limits were included in the study. Lung cancer screening data were identified from the prospective lung cancer screening registry housed within the EHR system. Baseline scans were defined as the first low-dose CT scan each patient completed, and all subsequent scans were classified as follow-up scans.

### Geocoding and geospatial analysis

Home addresses of screened individuals residing in Philadelphia at the time of their baseline scan were identified using addresses provided in the EHR, and residency status was confirmed by geocoding addresses using ArcGIS (27). Geocoding was done at the census tract level and for each system was performed separately and on site (28). After geocoding patient addresses, a spatial join was performed to determine the number of patients screened per census tract, Total patient counts and counts per census tract from each health system were then aggregated to calculate the total number of patients who resided in each census tract in Philadelphia across the lung cancer screening programs. To generate a screening rate, counts were normalized to 1000 age-eligible residents (50-79 years of age) in each census tract using US Census data (29). The amassed patient counts were joined with census tract–level data to visualize neighborhood characteristics and generate bivariate choropleth maps. The neighborhood-level variables defined a priori were adult smoking prevalence, lung cancer mortality rate per 1000 age-eligible residents, Yost Index, and tobacco retailer density per 1000 age-eligible residents. Descriptive statistical methods were used to summarize data in the tables. Data sources are described in the Supplementary Methods (available online) (14,30-36).

## Results

### Baseline characteristics and low-dose CT scan results

From a total cohort of 11 222 individuals receiving low-dose CT scans, 893 (8.0%) were excluded for lung cancer screening ineligibility. This study described 10 329 lung cancer screening–eligible individuals who resided in Philadelphia and underwent lung cancer screening at PLC2 member health systems with at least 1 low-dose CT scan during the study period (Table 1). Across the entire cohort, 48.1% of patients were non-Hispanic White, 36.6% were non-Hispanic Black, and 7.2% were Hispanic. Approximately 59.1% of individuals had a current smoking status, and 15.4% had a diagnosis of chronic obstructive pulmonary disease.

**Table 1.** Baseline characteristics of individuals screened for lung cancer

	All health systems		Jefferson Health		Penn Medicine		Temple Health	
	**N = 10** **329**		n = 4940		n = 1913		n = 3476	
	No.	%	No.	%	No.	%	No.	%
Age, y								
<55	176	1.70	74	1.50	5	0.26	97	2.79
55-59	2038	19.73	678	13.72	505	26.40	855	24.60
60-64	3296	31.91	1731	35.04	558	29.17	1007	28.97
65-69	2280	22.07	989	20.02	457	23.89	834	23.99
70-74	1954	18.92	1097	22.21	286	14.95	571	16.43
≥75	585	5.66	371	7.51	102	5.33	112	3.22
Sex								
Female	5394	52.22	2587	52.37	1019	53.27	1788	51.44
Male	4935	47.78	2353	47.63	894	46.73	1688	48.56
Race/ethnicity								
Non-Hispanic White	4972	48.14	3191	64.60	662	34.61	1119	32.19
Non-Hispanic Black	3781	36.61	1077	21.80	1140	59.59	1564	44.99
Hispanic	747	7.23	130	2.63	36	1.88	581	16.71
Other	395	3.82	164	3.32	55	2.88	176	5.06
Unknown	434	4.20	378	7.65	20	1.05	36	1.04
Insurance								
Medicare	4001	38.74	1081	21.88	1038	54.26	1882	54.14
Medicaid	1681	16.27	632	12.79	278	14.53	771	22.09
Private	2383	23.07	1226	24.82	360	18.82	797	22.93
Other^a^	2264	21.92	2001	40.50	237	12.39	26	0.75
Body mass index								
<25	2765	26.77	1219	24.68	579	30.27	967	26.41
25-29.9	3102	30.03	1467	29.70	617	32.25	1018	30.04
30-34.9	2076	20.10	859	17.39	434	22.69	783	22.53
35-39.9	1313	12.71	831	16.82	166	8.68	316	9.02
≥40	753	7.29	392	7.94	108	5.65	253	7.61
Missing	320	3.10	172	3.48	9	0.47	139	4.39
Chronic obstructive pulmonary disease	1587	15.36	1475	29.86	112	5.85	—^b^	—^b^
Personal history of cancer	404	3.91	368	7.45	36	1.88	—^b^	—^b^
Family history of lung cancer	935	9.05	739	14.96	—^b^	—^b^	196	5.64
Smoking status								
Current	6101	59.07	2906	58.83	1191	62.26	2004	57.65
Former	4228	40.93	2034	41.17	722	37.74	1472	42.35
Never	0	0.00	0	0.00	0	0.00	0	0.00
Missing	0	0.00	0	0.00	0	0.00	0	0.00
Smoking intensity, pack-years								
<30	1283	12.42	173	3.50	65	3.40	1045	30.06
30-49	4135	40.03	1994	40.36	1268	66.28	873	25.12
50-69	1938	18.76	1247	25.24	377	19.71	314	9.03
70-89	952	9.22	736	14.90	103	5.38	113	3.25
≥90	911	8.82	685	13.87	100	5.23	126	3.62
Missing	1110	10.75	105	2.13	0	0.00	1005	28.91

a Other insurance includes individuals with state marketplace and other plans, no health insurance, and missing insurance status.

b Data not collected.

Demographic characteristics differed among individuals screened in each health system. For example, race distribution differed among health systems, with the proportion of non-Hispanic Black individuals screened ranging from 21.8% to 59.6% and the percentage of Hispanic individuals ranging from 1.9% to 16.7%. Lung cancer risk factors, including chronic obstructive pulmonary disease (5.9% to 29.9%), personal history of cancer (1.9% to 7.5%), and family history of lung cancer (5.6% to 15.0%) were also different across health systems, although not all sites had data for these factors.

**Figure 1.**
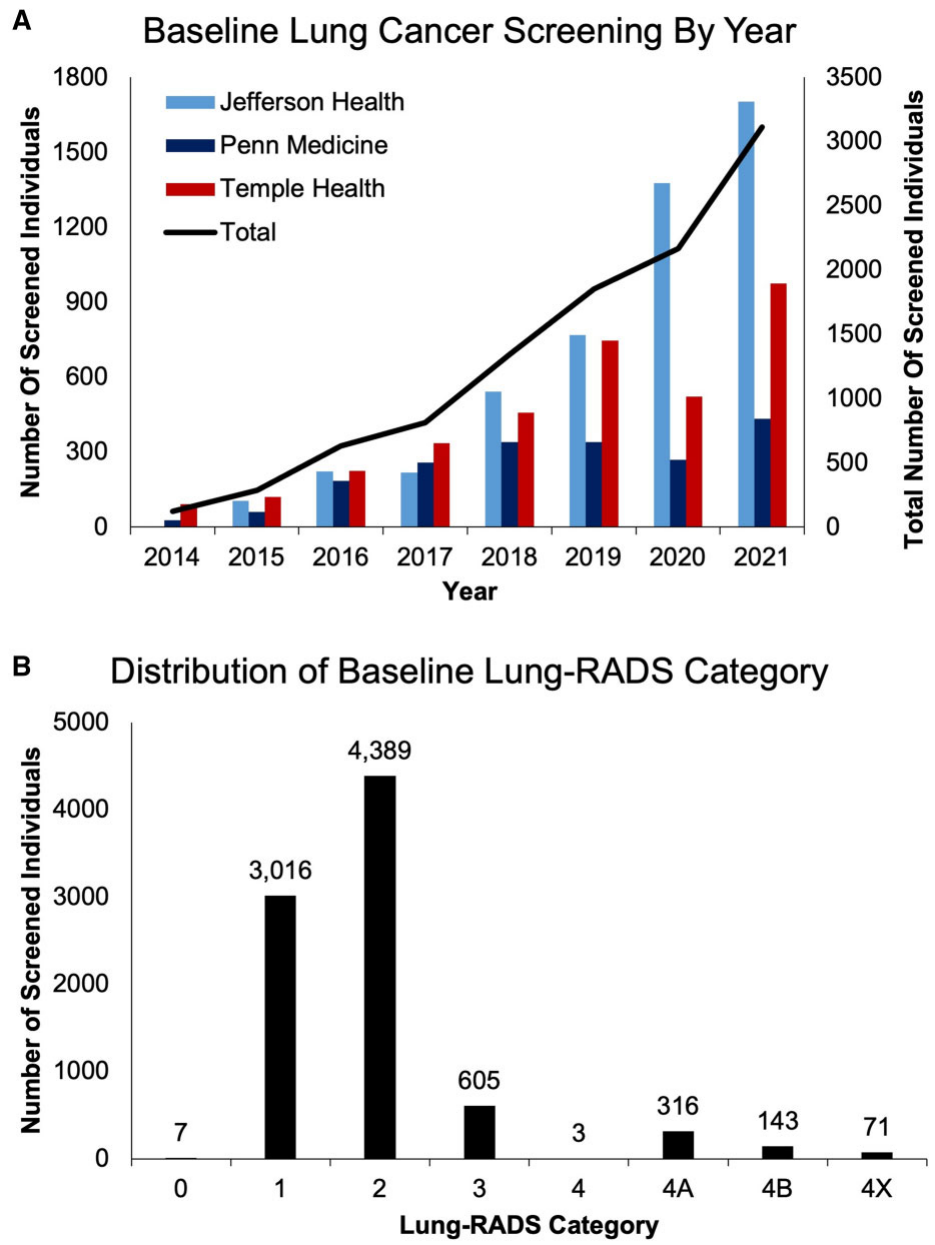
**Baseline lung cancer screening counts and results. A**) The number of individuals receiving a low-dose computed tomography scan for lung cancer screening per year across each Philadelphia Lung Cancer Learning Community health system (**bars**, left-sided *y*-axis) and all Philadelphia Lung Cancer Learning Community health systems combined (**line**, right-sided *y*-axis). **B**) Low-dose computed tomography results by Lung-RADS category across all Philadelphia Lung Cancer Learning Community health systems. One health system collected low-dose computed tomography results only for the most recent scan; therefore, not every scan has an assigned Lung-RADS score. Lung-RADS, Lung CT Screening Reporting & Data System.

The number of individuals receiving lung cancer screening increased from year to year, with 3111 individuals receiving baseline low-dose CT scans in 2021 (Figure 1). The most frequent low-dose CT scan result at baseline screening was Lung-RADS category 2. Eleven percent of individuals had a positive lung cancer screening result (Lung-RADS category 3 or higher).

### Citywide distribution of screened individuals

**Figure 2.**
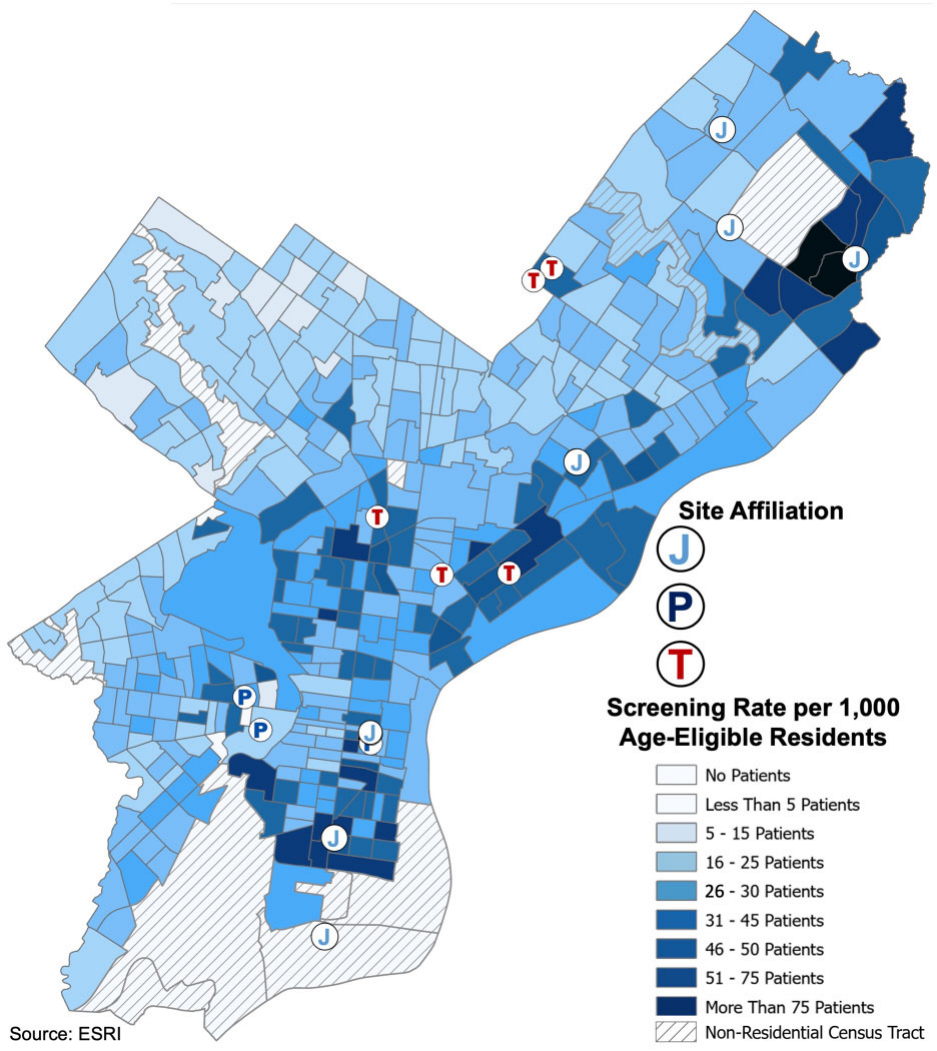
**Lung cancer screening rate per Philadelphia census tract.** Following geocoding, screening rates (patient count per 1000 age-eligible residents) in each Philadelphia census tract were merged across the 3 Philadelphia Lung Cancer Learning Community health systems. Lung cancer screening sites are denoted by **blue** (Jefferson Health), **navy** (Penn Medicine), and **red** (Temple Health) icons. Sources: ESRI, US Census.

Of the 374 residential census tracts in Philadelphia, 352 (94.1%) included at least 5 individuals undergoing lung cancer screening at 1 of the PLC2 member health systems (Supplementary Table 1, available online). The highest screening rates were in census tracts located in central and northeast Philadelphia and, to a lesser extent, in North and South Philadelphia (Figure 2). Census tracts with higher screening rates (>45 screened patients per 1000 age-eligible residents) were generally clustered around screening sites (Figure 2). Census tracts with lower screening rates (<45 screened patients per 1000 age-eligible residents) were located in upper North and northwest Philadelphia. Health system–specific analyses demonstrated geographic complementarity among the PLC2 institutions, with each health system primarily screening individuals residing in census tracts adjacent to their lung cancer screening sites.

### Neighborhood characteristics

**Figure 3.**
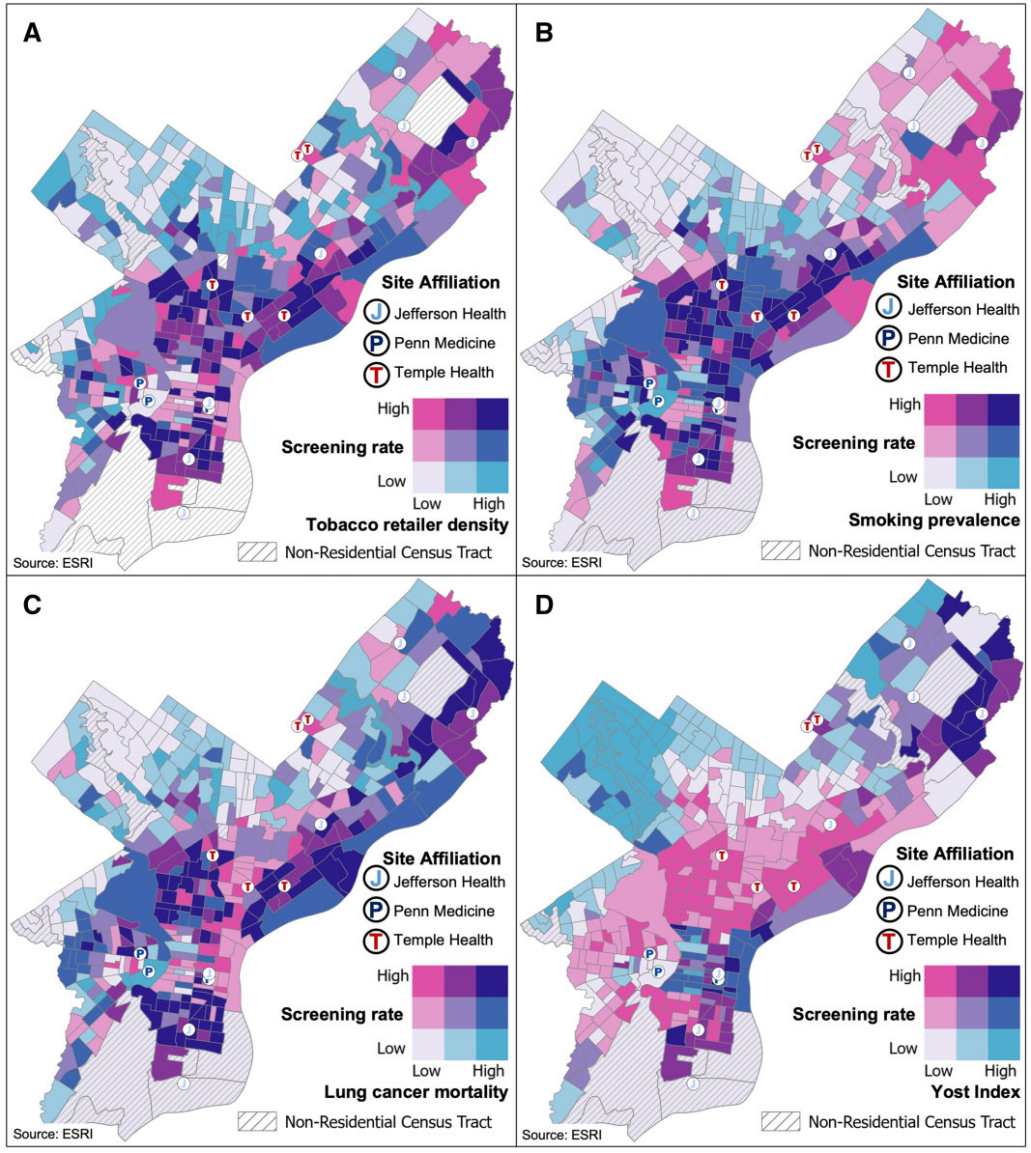
**Lung cancer screening rate and lung cancer–related factors per Philadelphia census tract.** Neighborhood-level data were mapped for each census tract in the city of Philadelphia. Measures included **A**) tobacco retailer density, **B**) adult smoking prevalence, **C**) lung cancer mortality, and **D**) Yost Index for socioeconomic status. A bivariate choropleth key is displayed for each panel, with screening rate (patient count per 1000 age-eligible residents) on the vertical axis and tertiles of lung cancer–related factors on the horizontal axis. Sources: ESRI, US Census, Public Health Management Corporation Community Health Database, Pennsylvania Cancer Registry, City of Philadelphia.

Bivariate choropleth maps were generated for Philadelphia census tract–level characteristics to analyze tobacco retailer density, smoking prevalence, lung cancer mortality, and socioeconomic status (Figure 3). Each map displays tertiles of screening rates (count per 1000 age-eligible residents, vertical axis on each map key) and a lung cancer–related factor (horizontal axis on each map key). In Figure 3, A and B, turquoise regions represent census tracts with high tobacco retailer density or high smoking prevalence, respectively, but low screening rates, with several of these high-risk census tracts clustered primarily in upper North Philadelphia. Similarly, regions of the upper North, northeast, and West Philadelphia had several census tracts with high lung cancer mortality but low screening rates, also in turquoise, as seen in Figure 3, C. Finally, much of the city consisted of census tracts with a low socioeconomic status, as represented in Figure 3, D by gray, light pink, and pink regions, particularly in North, West, and South Philadelphia. Taken together, this analysis demonstrates consistent areas of screening disparity across multiple measures, especially in North Philadelphia and West Philadelphia, where neighborhood-level lung cancer risk factors are elevated but screening rates are low.

Table 2 demonstrates the proportion of screened individuals stratified by Philadelphia census tract characteristics. Across all health systems, the majority of screened individuals resided in census tracts with the highest tobacco retailer density, with 58.3% to 76.0% of lung cancer screening participants coming from Philadelphia census tracts with more than 3 tobacco retailers per 1000 age-eligible residents. The distribution of the screened population between low and high tobacco retailer density census tracts varied among health systems, with nearly 20% of screened individuals residing in census tracts with fewer than 1 tobacco retailer per 1000 age-eligible residents at 1 health system. The distribution of screened individuals by census tract–level smoking prevalence was similar across all 3 PLC2 health systems, with 61.2% to 70.4% of lung cancer screening participants residing in census tracts with smoking prevalence up to 20% and 15.9% to 21.8% of residing in census tracts with the highest smoking prevalence (>30%). Analysis of the Yost Index by Philadelphia census tract revealed that among the screened population, 1 health system had a 75% greater proportion of individuals from census tracts with the greatest level of socioeconomic deprivation (quintile 1, 63.6% vs 35.8%) compared with another health system. Across all 3 health systems, more than half of individuals were residing in census tracts in the 2 most underserved quintiles of socioeconomic status, by Yost Index.

**Table 2.** Proportion of individuals screened for lung cancer, by site, stratified by Philadelphia census tract characteristics

	Overall cohort		Jefferson Health		Penn Medicine		Temple Health	
	N = 10 329		n = 4940		n = 1913		n = 3476	
	No.	%	No.	%	No.	%	No.	%
Tobacco retailer density								
<1/1000 (n = 52 census tracts [13.9%])^a^^,^^b^	1407	13.62	917	18.56	111	5.80	379	10.90
1/1000 (n = 44 census tracts [11.8%])	1142	11.06	562	11.38	202	10.56	378	10.87
2/1000 (n = 32 census tracts [8.6%])	1076	10.42	580	11.74	144	7.53	352	10.13
≥3/1000 (n = 246 census tracts [65.8%])	6700	64.87	2881	58.32	1454	76.01	2365	68.04
Smoking prevalence^c^								
<10% (n = 234 census tracts [60.9%])	2992	28.97	1545	31.28	614	32.10	833	23.96
10%-20% (n = 59 census tracts [15.4%])	3794	36.73	1933	39.13	565	29.53	1296	37.28
21%-25% (n = 13 census tracts [3.4%])	911	8.82	419	8.48	140	7.32	352	10.13
26%-30% (n = 15 census tracts [3.9%])	672	6.51	257	5.20	175	9.15	240	6.90
>30% (n = 55 census tracts [14.3%])	1956	18.94	786	15.91	417	21.80	753	21.66
Lung cancer mortality^b^								
<1/1000 (n = 2 census tracts [0.5%])	13	0.13	9	0.18	2	0.10	2	0.06
1-5/1000 (n = 12 census tracts [3.2%])	158	1.53	43	0.87	35	1.83	80	2.30
6-15/1000 (n = 153 census tracts [40.9%])	3350	32.43	1290	26.11	624	32.62	1436	41.31
16-25/1000 (n = 169 census tracts [45.2%])	5613	54.34	3044	61.62	1020	53.32	1549	44.56
>25/1000 (n = 38 census tracts [10.2%])	1191	11.53	554	11.21	230	12.02	407	11.71
Yost Index								
** **Quintile 1 (n = 184 census tracts [47.9%])	5065	49.04	1769	35.81	1086	56.77	2210	63.58
Quintile 2 (n = 69 census tracts [18.0%])	1986	19.23	1106	22.39	335	17.51	545	15.68
Quintile 3 (n = 44 census tracts [11.5%])	1369	13.25	833	16.86	194	10.14	342	9.84
Quintile 4 (n = 42 census tracts [10.9%])	1369	13.25	970	19.64	132	6.90	267	7.68
Quintile 5 (n = 28 census tracts [7.3%])	466	4.51	232	4.70	147	7.68	87	2.50
Quintile NA (n = 17 census tracts [4.4%])	70	0.68	30	0.61	17	0.89	23	0.66

a Number and percentage for each factor subcategory represent number and percentage of census tracts out of 376 residential census tracts in Philadelphia. NA = not assigned.

b Rate per 1000 age-eligible individuals per United States Preventive Services Task Force criteria (residents 50-79 years of age).

c Defined as “adults who have smoked at least 100 cigarettes in their lifetime and currently smoke ‘every day’ or ‘some days’” per Philadelphia Department of Public Health. Census tract–level rates were calculated using 2012 and 2018 Public Health Management Corporation Household Health Survey data to create more reliable estimates per the Philadelphia Department of Public Health methodology for city health assessment. Projection weights were used to estimate demographic data for populations living in the geographic entity (adult projection weight). Southeastern Pennsylvania Household Health Survey Public Health Management Corporation, Community Health Database (2018). Retrieved from http://CHDBDataPortal.phmc.org.

Screening distribution was similar among health systems with regard to census tract–level lung cancer mortality. The vast majority of individuals (85.9%-87.7%) were residing in census tracts with 6 to 25 lung cancer deaths per 1000 age-eligible residents, and approximately 11% of screened individuals were from census tracts with the highest lung cancer mortality.

## Discussion

This study presents a novel perspective on characterizing individuals who have undergone lung cancer screening at a citywide level, facilitated by a unique collaboration among the 3 largest health systems in Philadelphia. Our analysis demonstrates that the screened population in Philadelphia reflects some of the racial diversity of the city, but there are marked differences in health system–level and neighborhood-level characteristics. We found that screening counts are highest in census tracts directly surrounding screening sites, with each health system’s screening reach providing complementary coverage across much of the city. We also identified neighborhoods at risk based on tobacco retailer density, smoking prevalence, lung cancer mortality, and socioeconomic status, stratified by lung cancer screening counts per age-eligible population.

Equitable cancer screening is a critical part of reducing health disparities. The screened cohort consisted of 37% non-Hispanic Black individuals, which is nearly aligned with Philadelphia as a whole; the city consists of 40.1% non-Hispanic Black individuals (12), but given higher smoking rates among Black adults in our city (20.4% vs 17.5% among White individuals), we may still be underscreening this vulnerable population (12). We also observed relative underscreening of the Hispanic population, with just 7.2% in our cohort compared with 15.2% of Hispanic individuals in the city overall. Notably, Hispanic individuals have the highest smoking prevalence of all race and ethnic groups in Philadelphia, at 24.5% (12).

Like many large cities, health outcomes in Philadelphia vary by neighborhood. The life expectancy is lowest and the poverty rate is highest in census tracts located in North Philadelphia, which in our analyses was also characterized by higher rates of tobacco retailer density, smoking prevalence, lung cancer mortality, and disadvantaged socioeconomic status based on the Yost Index. The city of Philadelphia’s Community Health Assessment revealed that North Philadelphia residents have among the highest age-adjusted cancer mortality rates, at 246.8 cancer deaths per 100 000 people (14). Our analysis demonstrated that the distribution of lung cancer screening participants in high-risk census tracts (defined by our neighborhood-level lung cancer–related factors) was more alike than different among the PLC2 health systems. Future collaborative efforts to increase lung cancer screening uptake in Philadelphia—including providing education, improving access, and sharing resources—could be targeted to high-risk geographic areas, such as North Philadelphia and West Philadelphia. Finally, although the census tracts in each health system’s surrounding geographic area appear to be a major factor in defining a health system’s screened population, health-care organizations should have an obligation to provide equitable care across their entire catchment area, as reflected by the requirement for nonprofit hospitals to conduct community needs assessments (37). Given the potential impact that lung cancer screening can have in these communities, assessment of lung cancer screening and other preventive services should routinely include geospatial analyses such as these, measuring risk factors, disease outcomes, and measures of socioeconomic deprivation.

The neighborhood-level characteristics examined in this analysis are interrelated in their contribution to lung cancer risk. For instance, tobacco retailer density is associated with smoking prevalence, and both factors are in turn linked to neighborhood poverty rates (38-40). Additionally, neighborhood socioeconomic status contributes to racial and ethnic disparities in cancer-specific and overall survival (41). Although the Yost Index is a composite score, it is a validated measure in detecting socioeconomic gradients in cancer incidence and survival across racial groups (34,35). In a multi-institution analysis of patients receiving lung cancer screening, socioeconomic status, as measured by the Yost Index, accounted for nearly 50% of the observed racial disparity in annual lung cancer screening adherence (42). Disentangling the complex interplay of socioeconomic and environmental barriers is critical and should be the focus of future studies examining the impact of individual-level and neighborhood-level contextual factors (including language barriers and distance to screening centers) that may mediate lung cancer risk.

This study has several important limitations. First, the lung cancer screening program characteristics and data-collection methods differed at each health system. At Jefferson, individual-level data were either collected prospectively by the centralized lung cancer screening program or abstracted retrospectively through chart review, depending on the screening site, and at Penn and Temple lung cancer screening data were extracted from the EHR system using ordering and billing codes. Although these strategies introduce variability into the dataset and using aggregated data introduces the possibility of patients changing sites during subsequent rounds of screening and being counted twice in the dataset, this approach reflects real-world integration of data from multiple health systems. A second limitation is that although the PLC2 initiative consists of the 3 largest health systems in Philadelphia, no single hospital health system has a majority market share in the city, and many smaller hospitals, independent practice groups, and radiology sites also have overlapping catchment areas (43). Therefore, the screened cohort described in this study does not reflect the complete number of individuals undergoing lung cancer screening in Philadelphia. Finally, the lung cancer screening patients described in this study are characterized based on screening counts per census tract, normalized to age-eligible individuals, rather than as a percentage of USPSTF-eligible individuals. Although the latter measure would have greater immediate impact on targeting neighborhoods at risk for provision of lung cancer screening resources, it was outside the scope of this study to estimate the population eligible for lung cancer screening. Future studies from the PLC2 collaboration will focus on these analyses.

Previous geospatial analyses of lung cancer screening have used US Census data to estimate the lung cancer screening–*eligible* population in the context of access to high-quality lung cancer screening. Our analysis is unique in that it identified *screened* individuals at the local level, potentially facilitating citywide efforts to improve screening uptake. We estimate that the lung cancer screening uptake rate in Philadelphia may be approximately 12% (or 10 329 screened out of 89 231 eligible residents), although the accuracy of this projection is limited by the factors described here as well as by additional challenges in defining the eligible population (13). Compared with state-level data, however, this uptake rate is higher than Pennsylvania’s state rate of 9% and just below the top tier of states in the United States, which had uptake rates of 13% to 16.3% (4,44). These innovative concepts of integrating lung cancer screening data with geospatial analyses of lung cancer risk and local-regional collaborations among health systems can be broadly applied across the country. Future research should also focus on integrating these further analyses to identify neighborhoods in which residents experience underscreening, despite high lung cancer risk and lung cancer screening eligibility.

## Supplementary Material

pkad071_Supplementary_Data

## Data Availability

The data underlying this article are available in the article and in its online supplementary material.
