# Supplementary material for: The Philadelphia Lung Cancer Learning Community: a multi–health-system, citywide approach to lung cancer screening
Source: JNCI Cancer Spectr. 2023 Sep 15;7(5):pkad071. doi: 10.1093/jncics/pkad071 (PMC10588937; doi:10.1093/jncics/pkad071)
Supplement: pkad071_Supplementary_Data [file pkad071_supplementary_data.pdf]

SUPPLEMENTARY MATERIAL

SUPPLEMENTARY METHODS

Data Sources

Adult smoking prevalence was calculated using data from the Public Health Management Corporation's (PHMC) Household Health Survey (HHS).(29) The HHS is a population-representative survey conducted via telephone that gathers demographics, health risk factors and behaviors, as well as long-term health-related outcomes among residents of Southeastern Pennsylvania.(29) To reflect the demographics of Philadelphia's adult smoking population, PHMC employed projection weighting techniques to estimate demographic data for the population living in a specific geographic entity. This enabled an estimate of projected counts and percentages of adult smokers per census tract. Adult smokers were defined as "adults who have smoked at least 100 cigarettes in their lifetime and currently smoke "every day" or "some days" to align with the Philadelphia Department Of Public Health's Community Health Assessment.(14) Data from the 2012, 2015, and 2018 HHS were combined to create more reliable estimates per the Philadelphia Department Of Public Health's Community Health Assessment's methodology for adult smoking prevalence per planning district.(14,30,31)

Lung cancer mortality rate per 1,000 age-eligible residents was calculated utilizing lung cancer-related data points including, incidence and mortality for a ten-year period across the state of Pennsylvania, obtained from the Pennsylvania Cancer Registry. Lung cancer incidence was restricted to cases within Philadelphia city limits. Mortality rate per 1,000 residents was calculated by geocoding all lung cancer cases and deaths in the city, performing a spatial join to determine mortality per census tract, and adjusting rate per 1,000 residents of each census tract.

The Yost Index is a composite score of socioeconomic status used across state cancer registries based on principal component analysis of over forty census block groups, initially generated from breast cancer cases in the California Cancer Registry and 1990 US Census data.(32,33) This composite variable combines seven indicators of socioeconomic status: education, proportion with a blue-collar job, proportion older than 16 in the workforce without a job, median household income, proportion below 200% of the poverty level, median rent, and median house value.(34) The Yost Index provides two sets of census tract-level quintiles to assess socioeconomic status of each geographic unit. A state-based quintile allows for evaluation of census tracts within the same state. The United States-based quintile normalizes socioeconomic quintile scores to enable comparison across the country. We utilized the Yost US-based quintiles for this analysis to facilitate an equal comparison and increase generalizability; that is, allowing for a true comparison of socioeconomic status rank in each census tract in Philadelphia to the quintile rank of all other tracts in the United States. (33,34)

Each census tract was pre-designated with a score based on quintiles; where 1 indicates the lowest socioeconomic status and 5 is indicative of the greatest socioeconomic status. To provide a nationally representative perspective, "Yost Overall Quintile" was used in place of the state-specific ranking system.

Tobacco retailer density was calculated based on the comprehensive list of tobacco retailer permits in the city of Philadelphia.(35) The location of retailers were geocoded at the census tract-level, and a spatial join

was performed to establish total retailers per census rate, then rate of retailers per 1,000 age-eligible residents was calculated. Tobacco retailer density was calculated utilizing a comprehensive list of tobacco retailer permits issued by the city of Philadelphia in 2019 (n = 2,264 permits).(35) Each address associated with a permit was geocoded at the census tract-level. A spatial join was conducted in ArcGIS to establish the total number of retailers per census tract. After obtaining the aggregate number of vendors per census tract, tobacco retailer density per 1,000 age-eligible residents was calculated.

Supplementary Table 1. Patient Count and Screening Rate per Philadelphia Census Tract Merged

Census Tract	Total Patient Count n = 10,329		Non-Residential Tracts
	n	Rate Per 1,000 Age Eligible Residents	
Census Tract 353.02	114	81.72	
Census Tract 9.02	51	79.44	
Census Tract 353.01	121	75.86	
Census Tract 148	20	68.03	
Census Tract 348.03	99	67.72	
Census Tract 348.02	100	65.92	
Census Tract 11.01	53	63.55	
Census Tract 39.02	121	61.99	
Census Tract 33	111	60.52	
Census Tract 38	83	59.29	
Census Tract 24	58	58.23	
Census Tract 362.01	102	56.86	
Census Tract 363.03	106	55.58	
Census Tract 372	87	55.03	
Census Tract 362.02	114	54.52	
Census Tract 351	105	54.38	
Census Tract 40.01	48	53.87	
Census Tract 42.01	76	53.45	
Census Tract 173	38	52.27	
Census Tract 382	36	51.72	
Census Tract 39.01	107	50.47	
Census Tract 177.01	24	50.21	
Census Tract 362.03	83	49.82	
Census Tract 158	73	49.32	
Census Tract 174	34	48.71	
Census Tract 165	38	48.16	
Census Tract 18	24	47.71	
Census Tract 321	32	46.85	
Census Tract 109	26	46.68	
Census Tract 40.02	78	45.99	
Census Tract 179	53	45.34	
Census Tract 42.02	75	44.35	
Census Tract 147	11	44.18	
Census Tract 183	65	43.86	
Census Tract 363.01	57	43.68	
Census Tract 347.02	55	43.58	

Census Tract 133	21	42.94	
Census Tract 136.02	37	42.82	
Census Tract 365.02	55	42.54	
Census Tract 180.02	87	42.46	
Census Tract 27.02	57	42.10	
Census Tract 106	22	41.98	
Census Tract 293	28	41.67	
Census Tract 160	59	41.52	
Census Tract 169.02	53	41.12	
Census Tract 132	32	41.03	
Census Tract 32	49	40.77	
Census Tract 178	50	40.72	
Census Tract 151.01	25	40.58	
Census Tract 184	22	40.29	
Census Tract 331.02	35	39.19	
Census Tract 170	29	39.08	
Census Tract 157	20	38.91	
Census Tract 166	22	38.80	
Census Tract 199	34	38.59	
Census Tract 22	18	38.54	
Census Tract 28.02	54	38.38	
Census Tract 138	17	37.95	
Census Tract 188	61	37.58	
Census Tract 379	67	36.98	
Census Tract 92	19	36.96	
Census Tract 143	13	36.83	
Census Tract 28.01	21	36.52	
Census Tract 316	49	36.24	
Census Tract 9.01	19	36.12	
Census Tract 167.01	35	35.61	
Census Tract 168	39	35.58	
Census Tract 361	38	35.22	
Census Tract 339	34	35.12	
Census Tract 172.01	30	34.92	
Census Tract 300	62	34.79	
Census Tract 88.02	5	34.25	
Census Tract 245	35	34.01	
Census Tract 122.01	32	33.58	
Census Tract 131	12	33.43	
Census Tract 175	66	33.22	
Census Tract 323	29	33.11	
Census Tract 86.01	14	32.71	
Census Tract 137	47	32.59	
Census Tract 352	57	32.52	
Census Tract 153	20	32.00	
Census Tract 203	27	31.95	
Census Tract 348.01	58	31.94	
Census Tract 25	37	31.81	
Census Tract 180.01	30	31.48	
Census Tract 190	46	31.25	

Census Tract 41.02	64	31.16	
Census Tract 5	13	30.88	
Census Tract 298	39	30.54	
Census Tract 200	12	30.46	
Census Tract 36	64	30.26	
Census Tract 29	33	30.05	
Census Tract 30.02	21	29.79	
Census Tract 299	31	29.78	
Census Tract 163	25	29.76	
Census Tract 145	17	29.67	
Census Tract 156	14	29.66	
Census Tract 169.01	37	29.51	
Census Tract 151.02	36	29.27	
Census Tract 201.02	34	29.26	
Census Tract 11.02	13	29.21	
Census Tract 69	17	29.16	
Census Tract 152	44	29.16	
Census Tract 380	16	29.14	
Census Tract 177.02	29	29.09	
Census Tract 103	17	29.01	
Census Tract 171	41	29.00	
Census Tract 294	26	28.99	
Census Tract 91	22	28.76	
Census Tract 287	14	28.69	
Census Tract 141	26	28.57	
Census Tract 149	37	28.46	
Census Tract 318	28	28.31	
Census Tract 31	36	27.99	
Census Tract 146	20	27.97	
Census Tract 241	19	27.90	
Census Tract 202	46	27.85	
Census Tract 144	18	27.73	
Census Tract 107	24	27.59	
Census Tract 373	58	27.57	
Census Tract 74	34	27.55	
Census Tract 376	20	27.55	
Census Tract 16	14	27.50	
Census Tract 110	24	27.49	
Census Tract 41.01	42	27.31	
Census Tract 94	30	27.15	
Census Tract 4.01	22	27.13	
Census Tract 1	23	26.96	
Census Tract 363.02	29	26.93	
Census Tract 21	15	26.60	
Census Tract 10.02	32	26.58	
Census Tract 378	24	26.58	
Census Tract 66	23	26.56	
Census Tract 329	34	26.54	
Census Tract 136.01	23	26.41	
Census Tract 70	30	26.39	

Census Tract 192	48	26.23	
Census Tract 60	42	26.04	
Census Tract 2	14	26.02	
Census Tract 105	37	25.95	
Census Tract 27.01	23	25.93	
Census Tract 347.01	60	25.77	
Census Tract 381	6	25.75	
Census Tract 9800	1	25.64	
Census Tract 301	34	25.51	
Census Tract 81.02	34	25.49	
Census Tract 204	23	25.41	
Census Tract 30.01	24	25.37	
Census Tract 337.02	43	25.37	
Census Tract 167.02	25	25.25	
Census Tract 366	21	25.00	
Census Tract 139	17	24.89	
Census Tract 195.02	22	24.77	
Census Tract 325	44	24.66	
Census Tract 330	42	24.24	
Census Tract 334	39	24.16	
Census Tract 201.01	25	24.13	
Census Tract 239	15	24.12	
Census Tract 134.01	25	24.11	
Census Tract 242	27	23.87	
Census Tract 302	50	23.80	
Census Tract 383	18	23.75	
Census Tract 7	23	23.69	
Census Tract 197	48	23.68	
Census Tract 364	13	23.38	
Census Tract 345.01	33	23.34	
Census Tract 195.01	30	23.29	
Census Tract 319	30	23.24	
Census Tract 108	23	23.16	
Census Tract 336	48	23.01	
Census Tract 96	31	22.83	
Census Tract 93	30	22.78	
Census Tract 356.02	28	22.54	
Census Tract 198	39	22.38	
Census Tract 37.01	43	22.37	
Census Tract 55	43	22.37	
Census Tract 176.02	20	22.37	
Census Tract 243	23	22.29	
Census Tract 111	26	22.20	
Census Tract 161	26	22.17	
Census Tract 95	19	22.12	
Census Tract 162	13	22.03	
Census Tract 37.02	22	21.89	
Census Tract 172.02	33	21.87	
Census Tract 82	33	21.85	
Census Tract 331.01	28	21.82	

Census Tract 71.02	30	21.66	
Census Tract 9891	6	21.66	
Census Tract 320	36	21.56	
Census Tract 113	19	21.52	
Census Tract 4.02	39	21.38	
Census Tract 112	34	21.36	
Census Tract 215	18	21.28	
Census Tract 340	21	21.28	
Census Tract 67	43	21.09	
Census Tract 102	20	21.05	
Census Tract 191	43	20.92	
Census Tract 83.02	26	20.70	
Census Tract 17	14	20.68	
Census Tract 377	15	20.63	
Census Tract 367	15	20.49	
Census Tract 317	32	20.38	
Census Tract 61	16	20.33	
Census Tract 345.02	33	20.13	
Census Tract 73	17	19.88	
Census Tract 87.02	5	19.76	
Census Tract 15	16	19.61	
Census Tract 14	22	19.59	
Census Tract 56	4	19.51	
Census Tract 279.01	21	19.16	
Census Tract 305.02	33	19.08	
Census Tract 80	18	19.03	
Census Tract 83.01	29	18.99	
Census Tract 164	30	18.99	
Census Tract 342	22	18.98	
Census Tract 290	28	18.84	
Census Tract 314.02	22	18.80	
Census Tract 219	10	18.52	
Census Tract 246	14	18.49	
Census Tract 359	42	18.30	
Census Tract 356.01	40	18.29	
Census Tract 205	23	18.18	
Census Tract 10.01	23	18.10	
Census Tract 12.02	21	18.06	
Census Tract 20	10	17.95	
Census Tract 280	30	17.95	
Census Tract 23	18	17.86	
Census Tract 289.01	13	17.74	
Census Tract 6	4	17.70	
Census Tract 176.01	28	17.63	
Census Tract 305.01	17	17.62	
Census Tract 267	29	17.61	
Census Tract 140	13	17.54	
Census Tract 360	24	17.40	
Census Tract 135	17	17.38	
Census Tract 311.02	16	17.35	

Census Tract 207	21	17.16	
Census Tract 307	17	17.15	
Census Tract 84	23	17.10	
Census Tract 333	27	16.93	
Census Tract 292	20	16.89	
Census Tract 284	19	16.80	
Census Tract 142	11	16.72	
Census Tract 8.01	5	16.56	
Census Tract 326	31	16.49	
Census Tract 104	18	16.36	
Census Tract 357.01	25	16.36	
Census Tract 13	28	16.23	
Census Tract 3	15	16.20	
Census Tract 308	26	16.07	
Census Tract 346	13	16.07	
Census Tract 64	15	16.06	
Census Tract 212	10	16.00	
Census Tract 63	18	15.90	
Census Tract 85	35	15.86	
Census Tract 12.01	18	15.79	
Census Tract 282	27	15.70	
Census Tract 365.01	28	15.63	
Census Tract 332	11	15.56	
Census Tract 62	17	15.54	
Census Tract 247	24	15.49	
Census Tract 134.02	9	15.49	
Census Tract 119	28	15.42	
Census Tract 310	30	15.20	
Census Tract 283	31	15.11	
Census Tract 268	25	15.02	
Census Tract 125	18	14.99	
Census Tract 341	28	14.79	
Census Tract 262	23	14.75	
Census Tract 249	16	14.73	
Census Tract 81.01	10	14.56	
Census Tract 79	14	14.55	
Census Tract 279.02	9	14.38	
Census Tract 259	22	14.35	
Census Tract 210	17	14.30	
Census Tract 338	26	14.29	
Census Tract 220	9	14.26	
Census Tract 275	21	14.06	
Census Tract 313	27	13.85	
Census Tract 8.03	13	13.80	
Census Tract 258	10	13.76	
Census Tract 289.02	17	13.74	
Census Tract 270	13	13.67	
Census Tract 288	20	13.63	
Census Tract 208	7	13.54	
Census Tract 252	26	13.43	

Census Tract 72	18	13.38	
Census Tract 312	21	13.37	
Census Tract 71.01	10	13.18	
Census Tract 86.02	11	13.17	
Census Tract 98.02	22	13.17	
Census Tract 309	12	13.11	
Census Tract 311.01	15	13.10	
Census Tract 98.01	9	13.04	
Census Tract 218	13	13.03	
Census Tract 349	25	12.98	
Census Tract 240	14	12.83	
Census Tract 306	30	12.81	
Census Tract 244	9	12.75	
Census Tract 357.02	16	12.67	
Census Tract 122.04	19	12.66	
Census Tract 101	22	12.53	
Census Tract 344	41	12.44	
Census Tract 390	24	12.31	
Census Tract 114	25	12.30	
Census Tract 315.02	16	12.13	
Census Tract 274.01	16	12.02	
Census Tract 375	12	11.98	
Census Tract 355	34	11.95	
Census Tract 314.01	17	11.85	
Census Tract 276	15	11.83	
Census Tract 19	6	11.81	
Census Tract 272	20	11.70	
Census Tract 120	6	11.61	
Census Tract 263.01	17	11.53	
Census Tract 285	9	11.35	
Census Tract 271	12	11.25	
Census Tract 264	24	11.16	
Census Tract 278	15	11.10	
Census Tract 121	12	10.94	
Census Tract 369	2	10.64	
Census Tract 214	7	10.54	
Census Tract 65	15	10.52	
Census Tract 273	16	10.38	
Census Tract 281	11	10.34	
Census Tract 269	9	10.31	
Census Tract 358	28	10.26	
Census Tract 100	12	10.24	
Census Tract 9802	2	10.20	Non-Residential
Census Tract 291	10	10.17	
Census Tract 217	23	9.99	
Census Tract 265	13	9.83	
Census Tract 237	15	9.83	
Census Tract 87.01	4	9.71	
Census Tract 115	14	9.67	
Census Tract 337.01	19	9.64	

Census Tract 277	15	9.26	
Census Tract 238	14	9.25	
Census Tract 286	15	9.24	
Census Tract 274.02	16	9.09	
Census Tract 118	19	9.05	
Census Tract 335	10	9.03	
Census Tract 260	10	8.69	
Census Tract 213	7	8.66	
Census Tract 235	4	8.60	
Census Tract 315.01	15	8.39	
Census Tract 117	3	8.29	
Census Tract 54	4	8.06	
Census Tract 388	11	8.04	
Census Tract 8.04	10	7.90	
Census Tract 253	9	7.87	
Census Tract 389	9	7.81	
Census Tract 385	7	7.67	
Census Tract 206	3	6.90	
Census Tract 257	9	6.61	
Census Tract 236	6	6.42	
Census Tract 211	4	6.24	
Census Tract 384	6	6.12	
Census Tract 78	6	5.73	
Census Tract 386	4	5.46	
Census Tract 266	12	5.39	
Census Tract 216	3	4.54	
Census Tract 263.02	10	4.53	
Census Tract 255	4	4.39	
Census Tract 261	6	4.19	
Census Tract 254	6	4.08	
Census Tract 387	4	3.96	
Census Tract 248	2	3.75	
Census Tract 90	1	3.64	
Census Tract 256	4	3.48	
Census Tract 231	1	1.95	
Census Tract 209	1	1.93	
Census Tract 122.03	0	0.00	
Census Tract 77	0	0.00	
Census Tract 88.01	0	0.00	
Census Tract 9808	1	0.00	Non-Residential
Census Tract 9809	1	0.00	Non-Residential
Census Tract 9803	0	0.00	Non-Residential
Census Tract 9804	0	0.00	Non-Residential
Census Tract 9805	0	0.00	Non-Residential
Census Tract 9806	0	0.00	Non-Residential
Census Tract 9807	0	0.00	Non-Residential
Census Tract 9801	0	0.00	Non-Residential
Census Tract 50	0	0.00	Non-Residential
